# Development and Validation of a Novel Ferroptosis-Related Gene Signature for Predicting Prognosis and the Immune Microenvironment in Gastric Cancer

**DOI:** 10.1155/2021/6014202

**Published:** 2021-10-18

**Authors:** Feng Wang, Cheng Chen, Wei-Peng Chen, Zu-Ling Li, Hui Cheng

**Affiliations:** General Surgery, The People's Hospital of Binhai County, Jiangsu Province, China

## Abstract

Ferroptosis is a mode of regulated cell death that depends on iron and plays pivotal roles in regulating various biological processes in human cancers. However, the role of ferroptosis in gastric cancer (GC) remains unclear. In our study, a total of 2721 differentially expressed genes (DEGs) were filtered based on The Cancer Genome Atlas (TCGA) (*n* = 375) dataset. Weighted gene coexpression network (WGCNA) analysis was then used and identified 7 modules, of which the blue module with the most significant enrichment result was selected. By taking the intersections of the blue module and ferroptosis-related genes (FRGs), we obtained 23 common genes. Functional analysis was performed to explore the biological function of the genes of interest, and with univariate Cox regression (UCR) analysis, survival genes were screened to construct a prognostic model based on 3 genes (SLC1A5, ANGPTL4, and CGAS), which could play a role in predicting the survival of GC patients. UCR and multivariate Cox regression (MCR) analysis revealed that the prognostic index could be used as an independent prognostic indicator and validated using another GSE84437 dataset. Notably, patients in the high-risk group had higher mutation frequencies, such as TTN and TP53. TIMER analysis demonstrated that the risk score strongly correlated with macrophage and CD4+ T cell infiltration. In addition, the high- and low-risk groups illustrated different distributions of different immune statuses. Furthermore, the low-risk group had a higher immunophenoscore (IPS), which meant a better response to immune checkpoint inhibitors (ICIs). Finally, gene set enrichment analysis (GSEA) revealed several significant pathways involved in GC. In this study, a novel FRG signature was built that could predict GC prognosis and reflect the status of the tumor immune microenvironment.

## 1. Introduction

As a major public health issue globally, gastric cancer (GC) is the fourth leading cause of cancer-related death [1]. Because early stages of GC are usually asymptomatic, patients are diagnosed at an advanced stage, leading to poor survival [2]. Moreover, among patients with GC who receive adjuvant therapy, 50% experience local or distant disease recurrence [3]. Hence, the identification of reliable diagnostic and prognostic biomarkers is critical for GC, not only to improve prognostication but also to provide novel therapeutic targets for GC.

Ferroptosis is a newly characterized iron-dependent form of nonapoptotic-regulated cell death [4] triggered by lipid reactive oxygen species (ROS) [5]. Ferroptosis plays an important role in various tumor cells, such as fibrosarcoma, lung cancer, and prostate cancer cells [6–8]. In addition, several publications have reported that natural active components alleviate multidrug resistance in cancer and inhibit the progression of multiple tumors by inducing ferroptosis [9]. These findings suggest ferroptosis as a new player that regulates tumor-suppressive function. Nevertheless, prognostic models for FRGs have not been constructed for the prediction of overall survival (OS) in GC patients.

The present study performed comprehensive analyses utilizing TCGA and GEO. We evaluated the prognostic value of the FRGs and constructed a three-mRNA signature that could effectively predict patient survival in TCGA dataset and further validated this three-mRNA signature in the GEO dataset. Furthermore, we conducted functional studies on risk scores to elucidate the pathogenic mechanisms and provide a scientific basis for clinical diagnosis and treatment.

## 2. Materials and Methods

### 2.1. Patient Cohort and Data Preparation

RNA-seq transcriptome data, somatic mutation data, copy number variation, and the corresponding clinicopathological data were retrieved from TCGA database. (https://tcga-data.nci.nih.gov/tcga/) and GEO database (http://www.ncbi.nlm. http://nih.gov/geo/). TCGA data comprised 375 and 32 samples of GC tissues and adjacent normal tissues, respectively. By application of the GPL6947 platform (Illumina HumanHT-12 V3.0), GSE84437 contained 433 samples of GC tissues (Table 1). The FRGs were obtained from other research [10], which downloaded FRGs from the FerrDb website (http://www.zhounan.org/ferrdb/) and PubMed (https://pubmed.ncbi.nlm.nih.gov/).

### 2.2. Identification of DRGs

Differential expression of DRGs between tumor and normal samples was assessed using the R package limma [11]. The false discovery rate (FDR) < 0.05 and ∣log2(fold change) | >0.5 were visualized on boxplots and heatmaps using the “ggpubr” and “heatmap” packages, respectively.

### 2.3. Highly Coexpressed Gene Set–Gene Module

WGCNA was conducted by the WGCNA package [12] in R software. As a previous study showed that the WGCNA was sensitive to batch effects and outlier samples, we performed hierarchical cluster analysis [13]. The module eigengene (ME), which is considered representative of the gene expression profiles, was calculated to identify clinically associated modules based on DRGs. To identify the most tumor-related modules, we conducted module-trait relationship calculations for each module. Then, for genes in the significant tumor-related modules, we calculated the Gene Significance and Gene Module Membership (MM) within the genes, modules, and clinical traits. Finally, we identified the genes in the GC-related modules [14, 15].

### 2.4. Acquisition of Intersecting Genes

Overlapping genes were identified as candidates for the subsequent analysis and were oriented from the FRGs and WGCNA. The online tool was Draw Venn Diagram (http://bioinformatics.psb.ugent.be/webtools/Venn/). Coexpression analysis was performed using the “Corrplot” package.

### 2.5. Function and Pathway Enrichment Analyses

The clusterProfiler package in R [16] was used to test the statistical enrichment of functions. To assess functional categories, we used Gene Ontology (GO) and Kyoto Encyclopedia of Genes and Genomes (KEGG) pathway analyses. A *P* value of <0.05 and *q* value of <0.05 were set as the thresholds.

### 2.6. Construction of an FRG-Based Risk Score Model

The candidate FRGs were analyzed using UCR analysis (*P* < 0.05). The median values were defined as the cutoff values for high and low FRG expression in Cox survival analysis. After identifying the prognosis-related FRGs, we performed MCR analysis to identify independent prognostic FRGs. Finally, the risk score for each patient was calculated by taking the sum of the Cox regression coefficient for each signature gene multiplied by its corresponding expression value. The immunohistochemistry staining of genes was examined by The Human Protein Atlas (HPA) (https://www.proteinatlas.org/about/download). The time-dependent receiver operating characteristic (ROC) curve was plotted by the “survivalROC” R package.

### 2.7. Gene Set Enrichment Analysis (GSEA)

GSEA was performed using GSEA v4.0.3 software with 1000 permutations and weighted enrichment statistics. The median risk score was used as the cutoff point for high- or low-risk group classification [17].

### 2.8. Immune Infiltration Analysis

The infiltration level of immune cells in GC was predicted using the TIMER database [18]. Correlation analysis between six types of immune cells and risk score was then conducted (https://cistrome.shinyapps.io/timer/).

### 2.9. Exploring Relationships between Immune Components and Risk Groups

We used the CIBERSORT algorithm to estimate data on tumor-infiltrating immune cells. Violin plots were used to present the full distribution of the data [19].

### 2.10. Immunophenoscore Analysis

The immunophenoscore computed a score based on the gene expression values of immune-related genes into four classes: (1) effector cells, (2) immunosuppressive cells, (3) MHC molecules, and (4) selected immunomodulators [20], through which machine learning can derive the immunophenoscore of a patient without bias. The immunophenoscores of GC patients were obtained from TCIA (https://tcia.at/).

### 2.11. Statistical Analysis

For the analysis of differences between two groups, Student's *t*-test was performed. A Kaplan–Meier survival analysis was performed to estimate the survival curve. Pearson's correlation analyses were used to gauge the degree of correlation between certain variables. Statistical analyses were performed using the statistical software R (version 4.0.2). A value less than 0.05 (*P* < 0.05) was considered significant.

## 3. Results

### 3.1. Identification of DEGs

**Figure 1:**
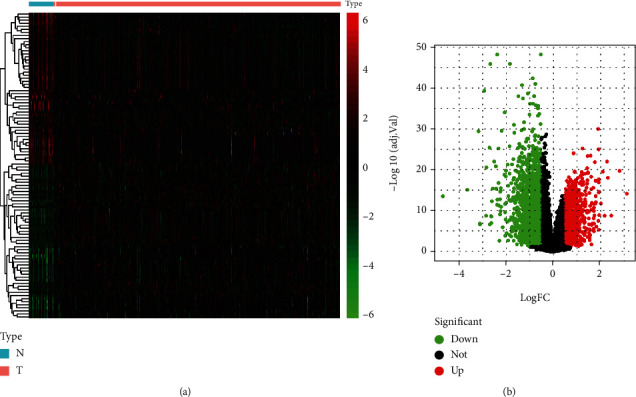
Differentially expressed genes (DEGs) obtained. (a) The heatmap of DEG expression level in GC samples. (b) The volcano plot of DEGs in GC. Red and blue indicate upregulation and downregulation, respectively. N: normal sample; T: tumor tissues.

After analysis, there were a total of 2721 DEGs between GC (*n* = 375) and normal samples (*n* = 32), including 1658 downregulated DEGs and 1063 upregulated DEGs. The heatmap (Figure 1(a)) and volcano plots (Figure 1(b)) are shown in Figure 1.

### 3.2. Identification of Significant Gene Modules by WGCNA

**Figure 2:**
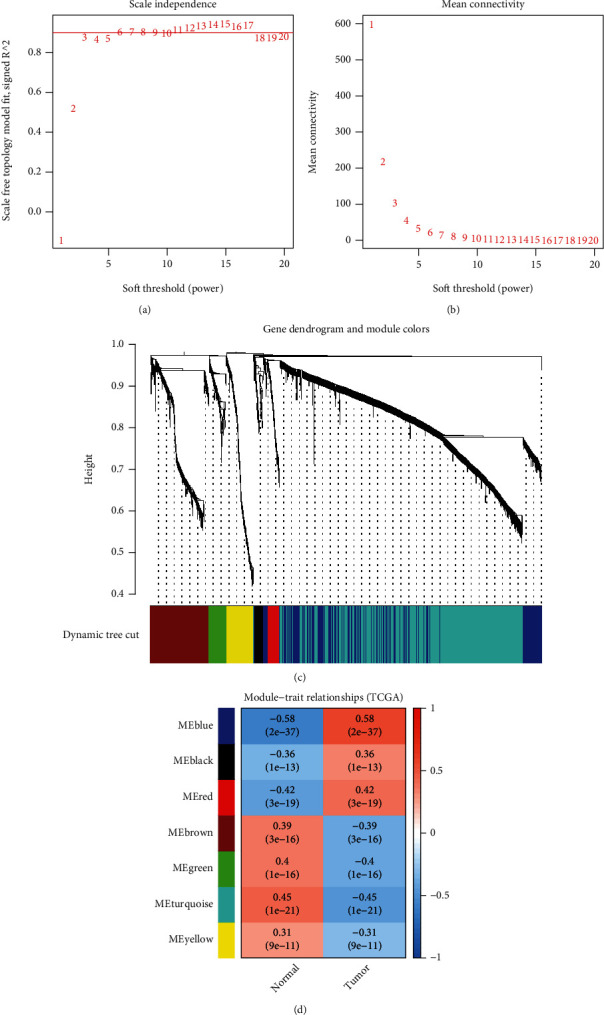
Identification of GC-related module genes by WGCNA. (a) The optimal power was 3 when the red guideline was set to 0.89. The mean connectivity of different soft-threshold powers. (b) Clustering of the dendrogram and corresponding modules. (c) Heatmap showing the correlation of gene modules with GC samples or normal samples. (d) Heatmap showing the correlation of gene modules with GC samples or normal samples.

**Figure 3:**
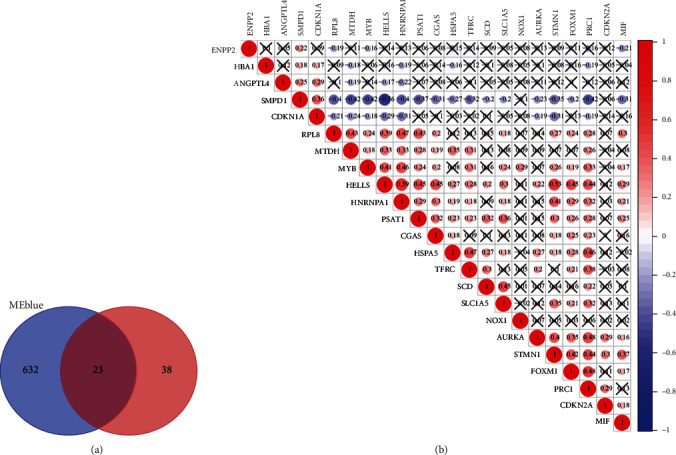
(a) Venn diagram displaying the number of genes in different groups. (b) Pearson's correlation analysis shows the relationship between the 23 FRGs based on their expression in GC tissues.

Overall, 2721 DEGs and 407 samples were selected after gene and sample screening and preprocessing. We used a power calculation of *β* = 3 (scale-free *R*^2^ = 0.895) (Figures 2(a) and 2(b)). There were 7 modules according to the network result (i.e., blue, black, red, brown, green, turquoise, and yellow modules). Among these 7 modules, the red (*r* = 0.42; *P* = 3*e* − 19), blue (*r* = 0.58; *P* = 2*e* − 37), and black (*r* = 0.36; *P* = 1*e* − 13) modules showed positive relationships with GC (Figures 2(c) and 2(d)). Furthermore, the genes in the turquoise and green modules showed strong negative correlations with GC (brown: *r* = −0.39; *P* = 3*e* − 16, green: *r* = −0.4; *P* = 1*e* − 16, turquoise: *r* = −0.45; *P* = 1*e* − 21, and yellow: *r* = −0.31; *P* = 9*e* − 11) (Figures 2(c) and 2(d)). Finally, we identified the blue module as the key module, in which there were 655 genes. Next, we compared the coexpressed genes in the blue module with the FRGs, and then a set of 23 shared genes was obtained (Figure 3(a)). Furthermore, there was a strong correlation among the FRGs (Figure 3(b)).

### 3.3. Functional Enrichment Analysis

**Figure 4:**
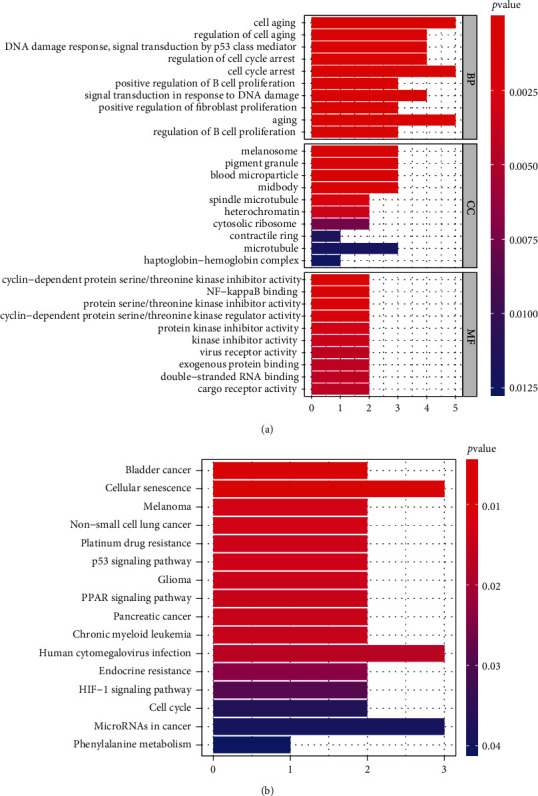
Gene functional annotation of the set of 23 shared genes. (a) GO analysis of the 23 shared genes, including biological process (BP), cellular component (CC), and molecular function (MF). (b) KEGG pathway enrichment analysis of the 23 shared genes. *P* < 0.05.

To better understand the signaling pathways and functions of FRGs in ferroptosis, functional enrichment of the 23 genes was performed, and FRGs were enriched in iron-related pathways, such as the regulation of cell aging, cell cycle arrest, and NF-kappaB binding. KEGG pathway analysis of the FRGs showed that genes were involved in ferroptosis, including cellular senescence, the p53 signaling pathway, phenylalanine metabolism, the HIF-1 signaling pathway, and the cell cycle (Figures 4(a) and 4(b)).

### 3.4. Construction of the Three-Gene-Based GC Prognostic Model

**Figure 5:**
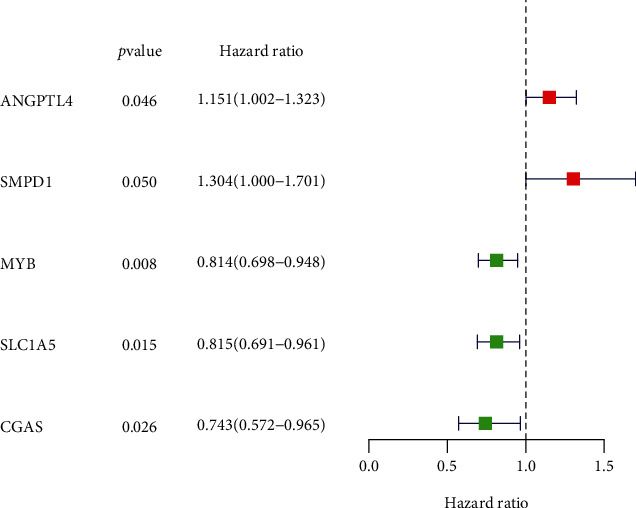
Screening prognostic factors through UCR analysis.

**Figure 6:**
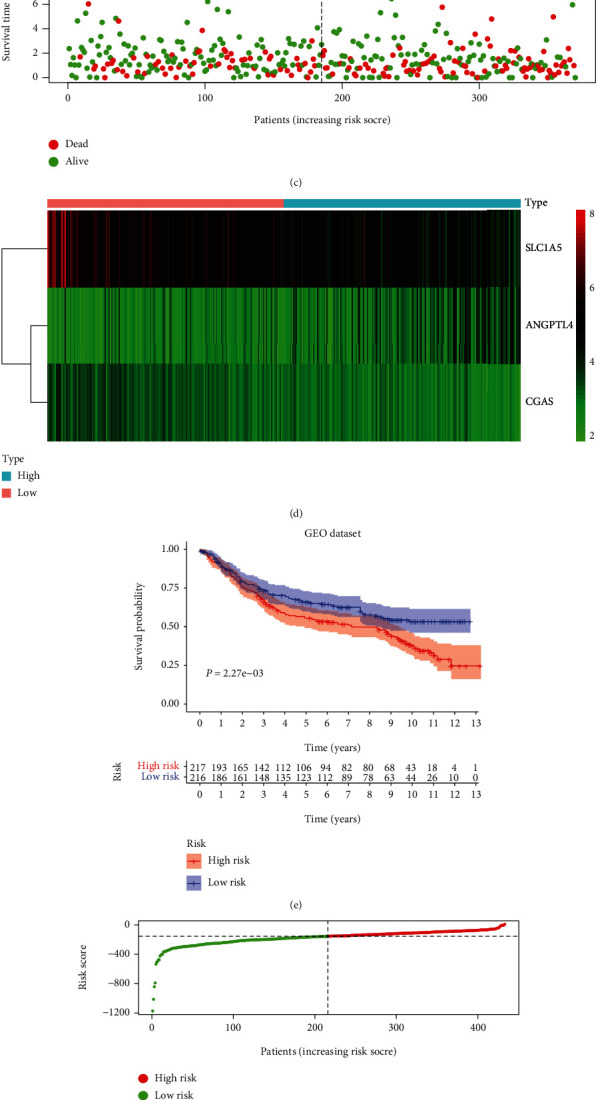
Prognostic model based on FRGs in GC. K-M survival analysis (a), risk score distribution (b), survival status (c), and heatmap (d) of a prognostic model in the GC cohort from TCGA. K-M survival analysis (e), risk score distribution (f), survival status (g), and heatmap (h) of a prognostic model in the GC cohort from GEO.

Then, UCR analysis of the screening results, including 23 FRGs, led to the identification of 5 FRGs as potential prognostic indicators of GC overall survival (OS), including ANGPTL4, SMPD1, MYB, SLC1A5, and CGAS (Figure 5). After primary filtering, we further shrunk the scope of gene screening. Three genes were identified: SLC1A5, ANGPTL4, and CGAS. To establish an optimal prognostic gene model, MCR analysis was performed on the three genes. The risk score was calculated by the following formula: risk score = [0.1497 × mRNA expression level of ANGPTL4] + [−0.1806 × mRNA expression level of SLC1A5] + [−0.2385 × mRNA expression level of CGAS]. After calculating the risk score, we divided 370 patients into the high-risk (*n* = 185) and low-risk (*n* = 185) groups using the median risk score as the cutoff. The patients in the high-risk group had worse OS than those in the low-risk group (Figure 6(a)). As the risk score rose, the patients had a shorter survival time and more deaths (Figures 6(b) and 6(c)). The risk heatmap showed the differences of three genes (SLC1A5, ANGPTL4, and CGAS) (Figure 6(d)). We used the GEO group for further external validation of this 3-gene-based signature. We got the same result as above (Figures 6(e)–6(h)). Next, the reliability and stability of the three gene-based models were further confirmed.

### 3.5. Assessment of Three FRG Signatures as Independent Prognostic Factors in GC Patients

**Figure 7:**
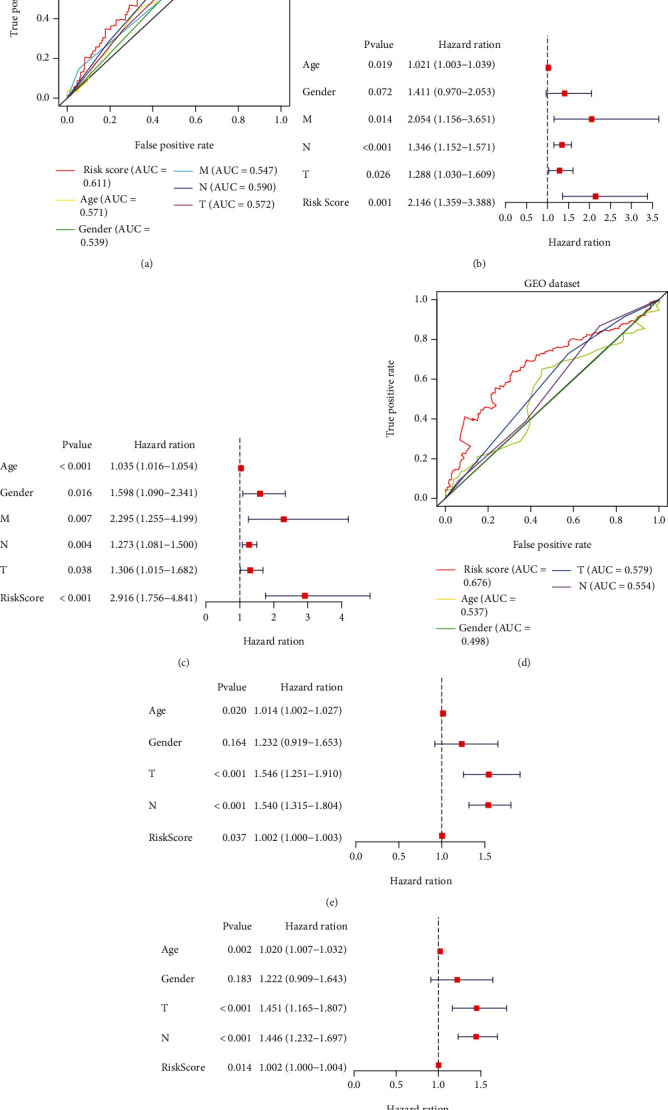
Prognostic value of risk subgroups in TCGA and GEO datasets. ROC curve (a), UCR (b), and MCR (c) analyses of the risk score and other clinical indices in the TCGA cohort. ROC curve (d), UCR (e), and MCR (f) analyses of the risk score and other clinical indices in the GEO cohort.

To further confirm whether the newly generated risk score was an independent risk factor in GC patients, we employed UCR and MCR analyses, which showed that T stage, N stage, metastasis, and risk score were independent prognostic factors for OS in GC (*P* < 0.001) (Figures 7(b) and 7(c)). To evaluate the diagnostic performance of the risk model in GC, ROC curves were constructed. The area under the ROC curve (AUC) of the risk score (0.611) was much higher than the AUC of age (0.571), sex (0.539), T stage (0.572), N stage (0.590), and metastasis status (0.547) (Figure 7(a)). All results illustrated that the three FRG signatures were independent prognostic factors in GC.

### 3.6. Validation of the Prognostic Performance of the FRG Signature in GC

**Figure 8:**
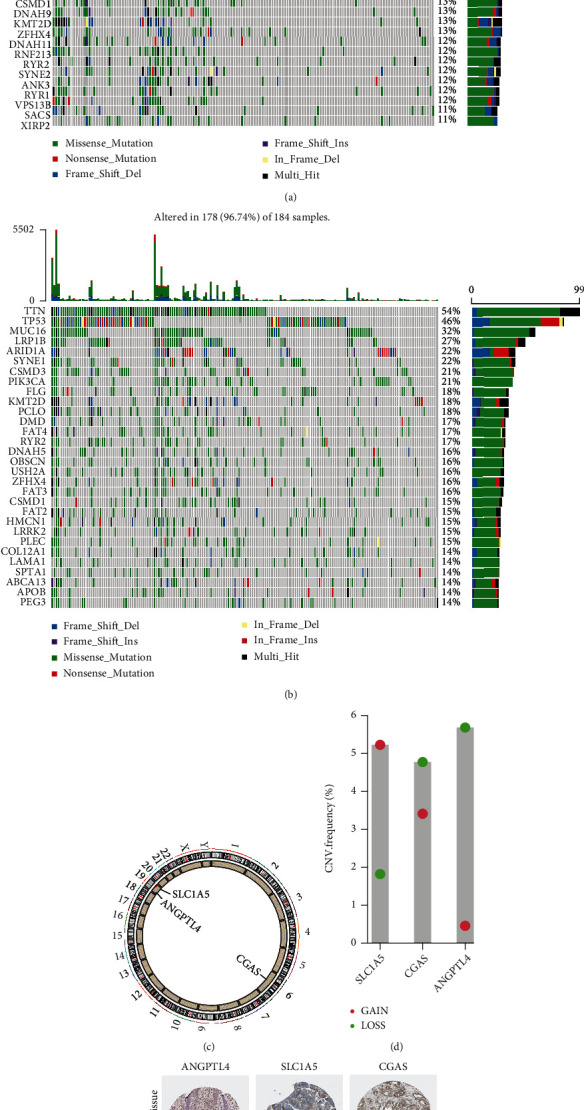
Genomic mutation profiles of different risk groups and genes. (a) Low-risk group and (b) high-risk group. The CNV mutation frequency (d) and location on chromosomes (c) of three genes. (e) The expression level of three genes in GC tissues.

To further assess outcome prediction, we combined the validation datasets (total of 433 patients) to evaluate the robustness of the three-gene signature. The results revealed the ROC curve AUC = 0.676 for validation datasets (Figure 7(d)), which is similar to the one in TCGA dataset. Cox regression analyses indicated that the risk score of the signature could be a powerful indicator of GC patient clinical outcome (Figures 7(e) and 7(f)). Interestingly, TTN [21], TP53 [22], MUC16 [23], and ARID1A [24] were the top mutations in both cohorts and were involved in various biological processes. In addition, the frequencies of all mutated genes were higher in the high-risk group (96.74%) (Figure 8(a)) than in the low-risk group (84.75%) (Figure 8(b)), suggesting that somatic mutation was positively correlated with risk scores. Further analysis of 3 signature genes revealed that CNV mutations were prevalent. SLC1A5 showed widespread CNV amplification. In contrast, ANGPTL4 and CGAS had prevalent CNV deletions (Figure 8(d)). The locations of CNV alterations of 3 signature genes on chromosomes are shown in Figure 8(c). Finally, the protein expression of SLC1A5, ANGPTL4, and CGAS was validated using the immunostaining results from the HPA database. As demonstrated in Figure 8(c), the SLC1A5 protein was highly expressed in GC tissue, while ANGPTL4 and CGAS were downregulated in GC.

### 3.7. Association between the Expression of 3 Signature Genes and Immune Markers

**Figure 9:**
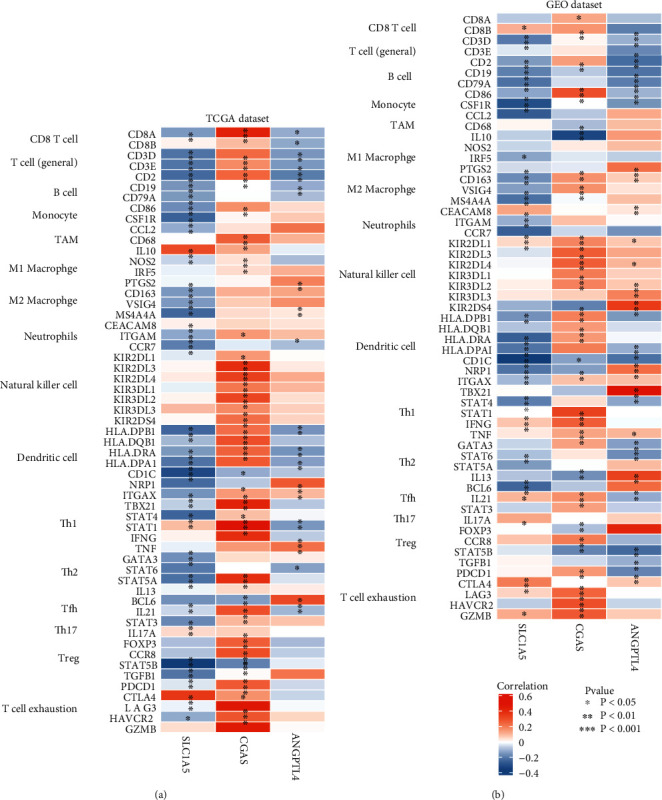
Correlation analysis between three genes and markers of immune cells in TCGA (a) and GEO (b) datasets.

Considering that ferroptosis was strongly associated with immune status, the correlations between the expression of 3 signature genes and immune markers were further explored in GC using TCGA and GEO datasets, including CD8+ T cells, T cells (general), and B cells. The expression level of CGAS showed a significant positive association with the level of most immune markers in T cells (general), natural killer cells, dendritic cells, Tregs, and T cell exhaustion (Figure 9(a)). Nonetheless, SLC1A5 and ANGPTL4 expression negatively correlated with immune markers, including B cells, T cells (general), and dendritic cells. Further reexamination using the GEO database revealed consistent results (Figure 9(b)).

### 3.8. Immune Profile and Response to Immune Checkpoint Inhibitors (ICIs) in Risk Groups

**Figure 10:**
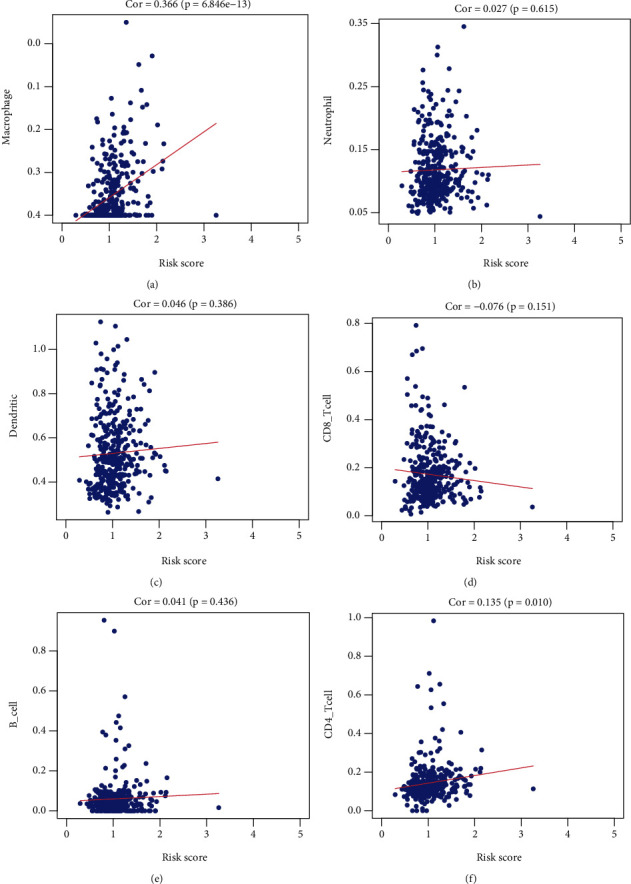
Correlation analysis between risk scores and immune cell infiltrations.

**Figure 11:**
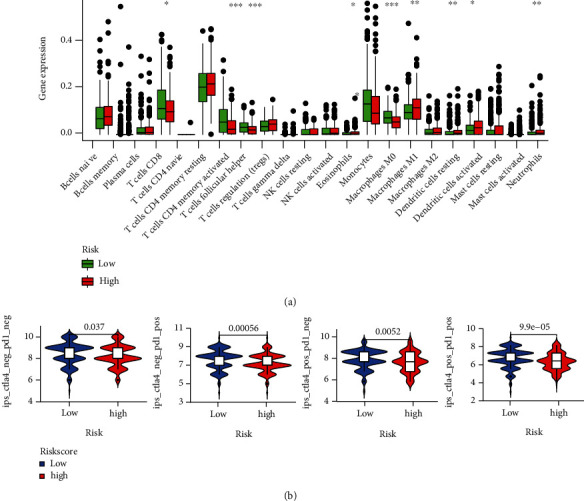
(a) Relative proportion of immune infiltration in high- and low-risk patients of the TCGA cohort. (b) Difference analysis of IPS between the low- and high-risk groups of the 3-gene model. ^∗^*P* < 0.05; ^∗∗^*P* < 0.01; ^∗∗∗^*P* < 0.005.

The correlations between risk scores and immune status were further explored using CIBERSORT and TIMER to evaluate the immune cell features. The risk score was positively associated with macrophages (*r* = 0.366; *P* = 6.846*e* − 13) and CD4+ T cells (*r* = 0.135; *P* = 0.010) (Figure 10). The results showed strong correlations between the ferroptosis-related risk model and the immunity of GC. As shown in Figure 11(a), monocytes, M2 macrophages, activated dendritic cells, resting mast cells, and neutrophils were upregulated in the high-risk group, while CD8+ T cells, activated CD4+ memory T cells, follicular helper T cells, and M2 macrophages were significantly downregulated (*P* < 0.05). Recent studies have revealed the role of the IPS in predicting the response to ICIs in melanoma patients based on high preexisting immunogenic potential [20]. Next, we investigated the relationship between IPS and the 3-FRG risk signature (Figure 11(b)). The results showed that IPS was significantly higher in the low-risk group than in the high-risk group (*P* < 0.05). Low-risk patients with the 3-FRG signature may have a better opportunity for ICI application. Moreover, the risk score was positively associated with macrophages (*r* = 0.366; *P* = 6.846*e* − 13) and CD4+ T cells (*r* = 0.135; *P* = 0.010) (Figure 10). The results showed strong correlations between the ferroptosis-related risk model and the immunity of GC.

### 3.9. Evaluation of Pathways within Both High- and Low-Risk TCGA Cohorts

**Figure 12:**
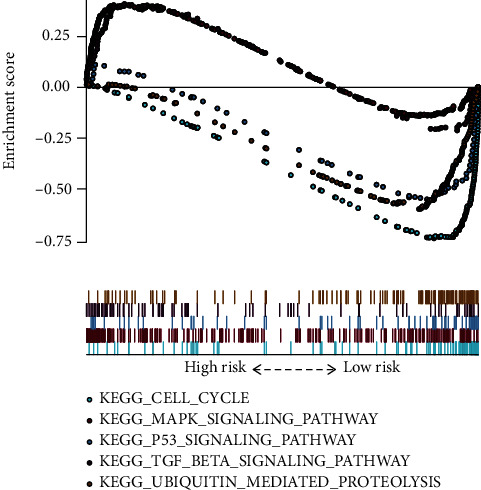
Functional enrichment analyses regarding the risk score.

GSEA was performed to identify gene sets differentially expressed in high- and low-risk groups from the MSigDB databases (c2.cp.kegg.v6.2.symbols.gmt). The cell cycle, P53, MAPK, ubiquitin-mediated proteolysis, and TGF-*β* signaling pathways were among the most significantly correlated enriched pathways (Figure 12; Table 2).

## 4. Discussion

Ferroptosis is an oxidative form of regulated cell death associated with the accumulation of lipid ROS because of enhanced lipid peroxidation [25, 26]. Increasing evidence suggests that ferroptosis plays a powerful role in enabling malignant features of tumors [27]. However, few studies have reported ferroptosis-related research in GC, and systematic analysis has not yet been elucidated.

In the present study, 23 differentially expressed FRGs between GC tissue and normal tissue were revealed by WGCNA and limma, and 5 FRGs of them were of prognostic value. Three gene prognostic models (SLC1A5, ANGPTL4, and CGAS) were constructed in TCGA dataset and validated in the GEO test set.

Solute carrier family 1 member 5 (SLC1A5), also referred to as ASCT2, is a sodium channel that acts as a high-affinity glutamine transporter in tumor cells [28]. Inhibition of SLC1A5 impedes glutamine uptake, leading to disturbance of mTORC1 signaling and activation of autophagy and cancer cell growth [29, 30]. Increased SLC1A5 expression has been documented in melanoma [31], neuroblastoma [32], and GC [33]. Previous research has shown that miR-137 suppresses ferroptosis by targeting the glutamine transporter SLC1A5 and decreases the antitumor activity of erastin in melanoma [34]. Angiopoietin-like 4 (ANGPTL4) is a member of the angiopoietin family, the members of which act as regulators of lipid and glucose metabolism [35]. ANGPTL4 is overexpressed in several types of cancers and is associated with poor clinical outcome [36, 37]. ANGPTL4 expression is related to cancer cell aggressiveness and migration [38, 39]. For instance, the expression of ANGPTL4 could be induced by TGF*β*, which could facilitate lung metastasis [38]. In addition, ANGPTL4 induces ROS accumulation and induces subsequent ferroptosis [40]. In addition, ANGPTL4 expression induces the resistance of ovarian cancer to carboplatin through ANGPTL4 [41]. Cyclic GMP-AMP synthase (CGAS) is a cytosolic DNA sensor that activates innate immune responses. cGAS catalyzes the synthesis of cGAMP, which functions as a second messenger that binds and activates the adaptor protein stimulator of interferon genes (STING) to induce type I interferons (IFNs) and other immune modulatory molecules [42]. The expression of cGAS, which produces cGAMP for STING activation, facilitates the activation of antitumor CD8+ T cell responses [43]. Furthermore, 8-hydroxy-2′-deoxyguanosine (8-OHG) functions as a damage-associated molecular pattern (DAMP) during ferroptotic cell death to trigger STING1-dependent macrophage polarization, supporting pancreatic cancer initiation and progression [44]. These results indicate that the three genes were closely related to tumor ferroptosis.

Considering the pivotal role of ferroptosis in the progression of tumor-invading immunity, immune cell infiltrations between low- and high-risk cohorts have also been discussed. A recent investigation documented that resting memory CD4 T cells are one of the most enriched tumor-invasive immune cells in GC samples [45]. Studies have reported follicular helper T cells in tertiary lymphoid structures of numerous tumors, implying that they participate in generating effective and sustained antitumor immune responses [46]. M1 macrophages are linked to antitumor activity, whereas M2 macrophages are associated with cancer progression and metastasis [45]. Herein, the high-risk group had an elevated level of M2 macrophages. In contrast, the patients in the low-risk group had elevated proportions of M1 macrophages. According to the IPS program, the results showed that IPS was significantly higher in the low-risk group, which indicated that low-risk patients with the 3-FRG signature had a better opportunity for ICI application. The GSEA collection found that the cell cycle, P53, MAPK, ubiquitin-mediated proteolysis, and TGF-*β* signaling pathways were most enriched. Recent studies have demonstrated the significant role played by p53 in the regulation of glucose metabolism, reactive oxygen species (ROS) responses, and ferroptosis [47]. Acetylation-defective p53 mutants were shown to promote ferroptosis, an iron-dependent, oxidative, and nonapoptotic form of cell death [47]. TGF-*β*1 is the most important cytokine of epithelial-to-mesenchymal transition (EMT), and tumor cells in a high state of oxidative stress have been reported to typically exhibit EMT, under which tumor cells may resist apoptotic cell death and increase their sensitivity to ferroptosis [48]. The mitogen-activated protein kinase (MAPK) signaling pathway has also been found to be involved in ferroptosis initiation [49]. Inhibiting MAPK signaling protects lung cancer cells against ferroptosis [50]. These GSEA results gave a detailed description of the ways and methods by which the three-gene signature participates in GC progression, which may benefit future medicine research.

The limitation of this study is that all data were obtained from public databases, and the adjacent normal samples were relatively small; there was a lack of experimental validation or a large sample size.

## 5. Conclusions

Our study found a novel, robust FRG signature for GC. The three-FRG signature can effectively evaluate GC patient prognosis and reflect the immune status. The three-FRG signature might be involved in the regulation of the immune-associated signaling pathway and might provide promising targets for improving prognosis and the responsiveness of GC to immunotherapy.

## Figures and Tables

**Table 1:** The clinical characteristics of the patients.

Items	Datasets		
	GSE84437		TCGA
Age	>65	150 (34.64%)	207 (55.05%)
	≤65	283 (65.36%)	164 (43.62%)
	NA	—	4 (1.06%)

Gender	Female	137(31.64%)	134 (35.64%)
	Man	296(68.36%)	241 (64.10%)

M	M0	—	330 (87.77%)
	M1	—	25 (6.65%)
	NA	—	20 (5.32%)

N	N0	80 (18.48%)	111 (29.52%)
	N1	188 (43.42%)	97 (25.80%)
	N2	132 (30.48%)	75 (19.95%)
	N3	33 (7.62%)	74 (19.68%)
	NA	—	18 (4.79%)

Stage	I	11 (2.54%)	19 (5.05%)
	II	38 (8.78%)	80 (21.28%)
	III	92 (21.25%)	168 (44.68%)
	IV	292 (67.44%)	100 (26.60%)
	NA	—	8 (2.13%)

NA: not applicable.

**Table 2:** Ferroptosis-related gene signature associated with the risk group.

Name	ES	NES	NOM *P* value	FDR *q* value
KEGG_TGF_beta_signaling_pathway	0.408	1.388	0.029	0.244
KEGG_MAPK_signaling_pathway	0.411	1.593	0.028	0.160
KEGG_cell_cycle	-0.749	-2.250	0.002	0.001
KEGG_P53_signaling_pathway	-0.567	-1.847	0.008	0.026
KEGG_ubiquitin_mediated_proteolysis	-0.605	-2.032	0.002	0.005

ES: enrichment score; NES: normalized enrichment score; NOM: nominal; FDR: false discovery rate.

## Data Availability

The datasets analyzed were acquired from The Cancer Genome Atlas (TCGA) database (https://portal.gdc.cancer.gov/) and GEO database (http://www.ncbi.nlm.nih.gov/geo/).

## Abbreviations

TCGA: The Cancer Genome Atlas
GEO: Gene Expression Omnibus
GC: Gastric cancer
WGCNA: Coexpression network analysis
FRGs: Ferroptosis-related genes
DEGs: Differentially expressed genes
DEFRGs: Differentially expressed ferroptosis-related genes
GO: Gene Ontology
KEGG: Kyoto Encyclopedia of Genes and Genomes
URC: Univariate Cox regression analyses
MCR: Multivariate Cox regression analyses
GSEA: Gene set enrichment analysis.

## References

[1] Siegel R. L., Miller K. D., Fuchs H. E. (2021). Cancer Statistics. *Jemal AJCACJfC: Cancer Statistics*.

[2] Hironaka S. (2019). Anti-angiogenic therapies for gastric cancer. *Asia-Pacific Journal of Clinical Oncology*.

[3] Niccolai E., Taddei A., Prisco D., Amedei A. (2015). Gastric cancer and the epoch of immunotherapy approaches. *World Journal of Gastroenterology*.

[4] Dixon S. J., Lemberg K. M., Lamprecht M. R. (2012). Ferroptosis: an iron-dependent form of nonapoptotic cell death. *Cell*.

[5] Zhao Y., Hu X., Liu Y. (2017). ROS signaling under metabolic stress: cross-talk between AMPK and AKT pathway. *Molecular Cancer*.

[6] Skouta R., Dixon S. J., Wang J. (2014). Ferrostatins inhibit oxidative lipid damage and cell death in diverse disease models. *Journal of the American Chemical Society*.

[7] Cao J. Y., Dixon S. J. (2016). Mechanisms of ferroptosis. *Cellular and Molecular Life Sciences*.

[8] Friedmann Angeli J. P., Schneider M., Proneth B. (2014). Inactivation of the ferroptosis regulator Gpx4 triggers acute renal failure in mice. *Nature Cell Biology*.

[9] Li Q. Y., Wang F., Yang X. C. (2019). Effects of times and storage conditions ofDuddingtonia flagranschlamydospores in sodium alginate pellets on its nematode predatory ability. *Biocontrol Science and Technology*.

[10] Liu Y., Zhang X., Zhang J., Tan J., Li J., Song Z. (2020). Development and validation of a combined ferroptosis and immune prognostic classifier for hepatocellular carcinoma. *Frontiers in Cell and Development Biology*.

[11] Ritchie M. E., Phipson B., Wu D. (2015). limma powers differential expression analyses for RNA-sequencing and microarray studies. *Nucleic Acids Research*.

[12] Langfelder P., Horvath S. (2008). WGCNA: an R package for weighted correlation network analysis. *BMC Bioinformatics*.

[13] Langfelder P., Horvath S. (2012). FastRFunctions for robust correlations and hierarchical clustering. *Journal of Statistical Software*.

[14] Oldham M. C., Konopka G., Iwamoto K. (2008). Functional organization of the transcriptome in human brain. *Nature Neuroscience*.

[15] Giulietti M., Occhipinti G., Principato G., Piva F. (2016). Weighted gene co-expression network analysis reveals key genes involved in pancreatic ductal adenocarcinoma development. *Cellular Oncology (Dordrecht)*.

[16] Yu G., Wang L. G., Han Y., He Q. Y. (2012). clusterProfiler: an R package for comparing biological themes among gene clusters. *OMICS*.

[17] Subramanian A., Tamayo P., Mootha V. K. (2005). Gene set enrichment analysis: a knowledge-based approach for interpreting genome-wide expression profiles. *Proceedings of the National Academy of Sciences*.

[18] Li T., Fan J., Wang B. (2017). TIMER: a Web server for comprehensive analysis of tumor-infiltrating immune cells. *Cancer Research*.

[19] Newman A. M., Liu C. L., Green M. R. (2015). robust enumeration of cell subsets from tissue expression profiles. *Nature Methods*.

[20] Charoentong P., Finotello F., Angelova M. (2017). Pan-cancer immunogenomic analyses reveal genotype-immunophenotype relationships and predictors of response to checkpoint blockade. *Cell Reports*.

[21] Liu Z., Wang L., Guo C. (2021). TTN/OBSCN 'Double-Hit' predicts favourable prognosis, 'immune-hot' subtype and potentially better immunotherapeutic efficacy in colorectal cancer. *Journal of Cellular and Molecular Medicine*.

[22] Chakraborty S., Martines C., Porro F. (2021). B cell receptor signaling and genetic lesions in TP53 and CDKN2A/CDKN2B cooperate in Richter transformation. *Blood*.

[23] Shen H., Guo M., Wang L., Cui X. (2020). MUC16 facilitates cervical cancer progression via JAK2/STAT3 phosphorylation-mediated cyclooxygenase-2 expression. *Genes Genomics*.

[24] Odnokoz O., Wavelet-Vermuse C., Hophan S., Bulun S., Wan Y. J. E. (2021). ARID1 proteins: from transcriptional and post-translational regulation to carcinogenesis and potential therapeutics. *Epigenomics*.

[25] Stockwell B. R., Friedmann Angeli J. P., Bayir H. (2017). Ferroptosis: a regulated cell death nexus linking metabolism, redox biology, and disease. *Cell*.

[26] Alvarez S. W., Sviderskiy V. O., Terzi E. M. (2017). NFS1 undergoes positive selection in lung tumours and protects cells from ferroptosis. *Nature*.

[27] Liang C., Zhang X., Yang M., Dong X. J. A. (2019). Recent progress in ferroptosis inducers for cancer therapy. *Advanced Materials*.

[28] Kanai Y., Hediger M. (2004). The glutamate/neutral amino acid transporter family SLC1: molecular, physiological and pharmacological aspects. *Pflügers Archiv*.

[29] Nicklin P., Bergman P., Zhang B. (2009). Bidirectional transport of amino acids regulates mTOR and autophagy. *Cell*.

[30] Hassanein M., Hoeksema M., Shiota M. (2013). SLC1A5 mediates glutamine transport required for lung cancer cell growth and survival. *Clinical Cancer Research*.

[31] Wang Q., Beaumont K. A., Otte N. J. (2014). Targeting glutamine transport to suppress melanoma cell growth. *International Journal of Cancer*.

[32] Ren P., Yue M., Xiao D. (2015). ATF4 and N-Myc coordinate glutamine metabolism inMYCN-amplified neuroblastoma cells through ASCT2 activation. *The Journal of Pathology*.

[33] Niu Y., Zhang J., Tong Y., Li J., Liu B. (2019). Physcion 8-O-*β*-glucopyranoside induced ferroptosis via regulating miR-103a-3p/GLS2 axis in gastric cancer. *Life Sciences*.

[34] Luo M., Wu L., Zhang K. (2018). miR-137 regulates ferroptosis by targeting glutamine transporter SLC1A5 in melanoma. *Cell Death and Differentiation*.

[35] Hato T., Tabata M., Oike Y. (2008). The role of angiopoietin-like proteins in angiogenesis and metabolism. *Trends in Cardiovascular Medicine*.

[36] Baba K., Kitajima Y., Miyake S. (2017). Hypoxia-induced ANGPTL4 sustains tumour growth and anoikis resistance through different mechanisms in scirrhous gastric cancer cell lines. *Scientific Reports*.

[37] Liao Y. H., Chiang K. H., Shieh J. M. (2017). Epidermal growth factor-induced ANGPTL4 enhances anoikis resistance and tumour metastasis in head and neck squamous cell carcinoma. *Oncogene*.

[38] Padua D., Zhang X. H., Wang Q. (2008). TGF*β* Primes Breast Tumors for Lung Metastasis Seeding through Angiopoietin- like 4. *Cell*.

[39] Li H., Ge C., Zhao F. (2011). Hypoxia-inducible factor 1 alpha-activated angiopoietin-like protein 4 contributes to tumor metastasis via vascular cell adhesion molecule-1/integrin *β*1 signaling in human hepatocellular carcinoma. *Hepatology*.

[40] Yang W.-H., Huang Z., Wu J., Ding C.-K. C., Murphy S. K., Chi J.-T. (2020). A TAZ-ANGPTL4-NOX2 axis regulates ferroptotic cell death and chemoresistance in epithelial ovarian cancer. *Molecular cancer research: MCR*.

[41] Zhou S., Wang R., Xiao H. (2020). Adipocytes induce the resistance of ovarian cancer to carboplatin through ANGPTL4. *Oncology Reports*.

[42] Wang H., Hu S., Chen X. (2017). cGAS is essential for the antitumor effect of immune checkpoint blockade. *Proceedings of the National Academy of Sciences of the United States of America*.

[43] Schadt L., Sparano C., Schweiger N. A. (2019). Cancer-cell-intrinsic cGAS expression mediates tumor immunogenicity. *Cell Reports*.

[44] Liu J., Dai E., Kang R., Kroemer G., Tang D. (2021). The dark side of ferroptosis in pancreatic cancer. *Oncoimmunology*.

[45] Gambardella V., Castillo J., Tarazona N. (2020). The role of tumor-associated macrophages in gastric cancer development and their potential as a therapeutic target. *Cancer Treatment Reviews*.

[46] Gu-Trantien C., Loi S., Garaud S. (2013). CD4+ follicular helper T cell infiltration predicts breast cancer survival. *The Journal of Clinical Investigation*.

[47] Jiang L., Kon N., Li T. (2015). Ferroptosis as a p53-mediated activity during tumour suppression. *Nature*.

[48] Yang W., SriRamaratnam R., Welsch M. (2014). Regulation of ferroptotic cancer cell death by GPX4. *Cell*.

[49] Chang W.-T., Bow Y.-D., Fu P.-J. (2021). A Marine Terpenoid, Heteronemin, Induces Both the Apoptosis and Ferroptosis of Hepatocellular Carcinoma Cells and Involves the ROS and MAPK Pathways. *Oxidative Medicine and Cellular Longevity*.

[50] Poursaitidis I., Wang X., Crighton T. (2017). Oncogene-selective sensitivity to synchronous cell death following modulation of the amino acid nutrient cystine. *Cell Reports*.

[51] Wang F., Chen C., Chen W.-P., Li Z.-L., Cheng H. (2021). Identification the ferroptosis-related gene signature in gastric cancer based on weighted gene co-expression network analysis (WGCNA).

